# The Pragmatics and the Normativity of Ignorance Attributions

**DOI:** 10.1007/s11098-026-02502-4

**Published:** 2026-03-13

**Authors:** Giorgia Foti

**Affiliations:** Department of Philosophy, Cogito Epistemology Research Centre, University of Glasgow, 67-69 Oakfield Avenue, Glasgow, G120HH UK

**Keywords:** Ignorance attributions, Normative Account, Conversational implicature, Communicative functions

## Abstract

The way we attribute ignorance to each other is often connected to normative considerations. Intuitively, attributions such as ‘S is ignorant that *p*’ sound odd when S is under no obligation to know that *p*. These linguistic intuitions about attributions of ignorance have been used to motivate the increasingly popular Normative Account of the nature of ignorance (Pritchard 2021a, 10.5840/philtopics202149223 2021b; 10.1007/s10670-022-00529-72024) according to which ignorance is a lack of a positive epistemic standing which manifests a failure of inquiry. Here, I will argue that champions of the Normative Account who want to draw metaphysical and conceptual conclusions about ignorance from linguistic intuitions may be too quick to do so. In fact, the normative weigh of ignorance attributions can be explained on a pragmatic basis.

## Introduction

Traditionally, ignorance has been identified simply with the lack of a positive epistemic state. Recent epistemology has focused on the normative dimension of this notion. According to the increasingly popular^1^ Normative account (Meylan, 2020, 2024; Pritchard, 2021a, 2021b) ignorance doesn’t just amount to an absence, but it is a lack of a positive epistemic standing which manifests a failure of proper inquiry. One key motivation for this new account comes from linguistic intuitions about how we attribute ignorance to each other (Pritchard, 2021a, 2021b). In a nutshell, as the argument goes, ascriptions of ignorance always entail a negative appraisal of the subject, and this is best explained by positing that ignorance is a lack of a positive epistemic standing which the agent ought to have.

This paper aims to undermine the above Argument from linguistic data in support of the Normative Account of ignorance, by explaining away the underlying intuitions on a pragmatic level. Drawing on the communicative functions of ignorance ascriptions, I will argue that the perceived oddity of ignorance attributions in cases where the agent has not failed as an inquirer should be traced back to the truth-conditions of the proposition pragmatically imparted by ignorance ascriptions in some stereotypical conversational contexts. In fact, attributions of ignorance that *p* may trigger the implicature that the agent ought to know that *p*, when they are used to express criticism towards the agent’s epistemic performance. While in this kind of conversational context the norms of inquiry coincide with the normative expectations generated by the conversational maxims, the same doesn’t hold for other kinds of conversational contexts where ignorance ascriptions have a different communicative function. Ultimately, the normative import of ignorance attributions pertains to their warranted assertability^2^.

If my explanation proves convincing, the Normative Account of ignorance would lose one key advantage over non-normative views, according to which ignorance is simply the absence of a positive epistemic state—whether knowledge (as argued by Le Morvan, 2011a, 2011b)^3^ or true belief (as argued by Peels, 2023)^4^. Relatedly, if one accepts my pragmatic explanation of the normative weight carried by ignorance ascriptions, one avoids the burden of developing an error theory for attributions of ignorance that lack such weight—namely, cases of blameless ignorance^5^, ignorance ascriptions with exculpatory force, and self-ascriptions of ignorance.

The discussion will proceed as follows. In the Sect. 2, after some preliminary remarks, I will outline the linguistic data about ignorance attributions which motivate the Normative Account, and I will further clarify what the explanation of these data amounts to. The third section will be devoted to my positive proposal: a pragmatic explanation of the normativity of ignorance attributions. I will start by outlining some plausible communicative functions of ignorance ascriptions, and I will put emphasis on the fact that only *some* of them are normative, in the sense that matters for the Normative Account^6^, namely tracking failure of proper inquiry. I will then argue that ‘being the result of a failure of inquiry’ is a conversational implicature of attributions of ignorance which play a specific subset of communicative functions, which make so that the norms of inquiry coincide with the normative expectations of the conversational context. This will pave the way to providing a pragmatic explanation of the linguistic data. I will then proceed in § 4 to address two preliminary objections which may be moved against my view: the objection from the difference between attributions of ignorance and negative attributions of knowledge and the objection from the gradability of ignorance and comparative ignorance attributions. I will argue that none of these objections succeeds. Finally, in § 5 I will situate the upshot of my project within the growing literature in the epistemology of ignorance, and I will suggest that there may be more to be gained from keeping ‘ignorance’ as a non-normative, umbrella term (Piedrahita, 2025b).

## The argument from linguistic data in support of the Normative Account

Before turning to the details of the argument, let me briefly clarify the kind of ignorance at issue here and pre-empt a couple of preliminary worries about the linguistic data which will be discussed. Ignorance can come in many different forms: one can be ignorant of the existence of Maria’s new dog because one has never met it (objectual ignorance); one can be ignorant of how to cook parmigiana (practical ignorance or ignorance-how)^7^. Many people are ignorant *that* Santa Venerina is a town in Italy (propositional or factual ignorance^8^). I am ignorant of both quantum physics and the poetics of neorealist cinema (topical ignorance^9^). Finally, one can be ignorant of the right answer to the test, or of how many people live in Barcelona, or of which of the suspects is the perpetrator (erotetic ignorance). The latter two can be both reduced to ignorance of propositions: the set of true propositions which constitute a topic in the case of topical ignorance and the true proposition which constitutes the answer to a given question in the case of erotetic ignorance^10^. In this paper, I will be concerned with attributions of factual ignorance^11^, typically expressed by the predicate ‘is ignorant’ and a declarative complement. Statements which express propositional ignorance will thus take the following form: ‘S is ignorant *that p*’ and ‘S is ignorant *of the fact that p*’.

Regarding these constructions, two worries need to be addressed. Firstly, it has been pointed out that the construction ‘ignorant that-’ sounds archaic and/or ungrammatical^12^. While I do not take a stance on this matter, I think this worry does not undermine the significance of my project. In fact, no matter whether the construction is grammatical or not, it is used by ordinary English speakers as the examples surveyed by Siscoe (2024) show^13^. Moreover, this construction is widely used in the literature on the epistemology of ignorance, where my project is situated. However, since I think the second construction ‘ignorant of the fact that *p*’ may get us closer to ordinary language while preserving the philosophical significance of the examples^14^, I will use both constructions interchangeably in the rest of the paper.

Secondly, one may worry that attributions of ‘ignorant that *p*’ seem to presuppose or conversationally imply that the attributor knows or at least believes that *p* is true^15^. This seems to apply *a fortiori* to the construction ‘ignorant of the fact that’, in which the declarative complement is a true proposition or a fact. The same doesn’t seem to apply to constructions such as ‘ignorant whether-’ or ‘ignorant as to whether-’, which express ignorance with respect to the truth value of a proposition (that can happen to be false). Not much hangs on this difference here, since I will remain neutral as to whether it is possible to be ignorant of false propositions and since I take erotetic ignorance to be reducible to propositional ignorance^16^. However, since I don’t want to play with the readers’ linguistic intuitions when discussing the cases and since the argument from linguistic intuitions is standardly framed in terms of ‘truths’, I will generally prefer the grammatical construction ‘ignorant that-’, and ‘ignorant of the fact that-’ and I will put ‘ignorant whether-’ among parentheses only when appropriate.

The Argument from linguistic data starts from the premise that there are cases in which the agents count as propositionally ignorant, according to the available accounts of ignorance as lack of knowledge (Le Morvan, 2011a, 2011b; 2013) or true belief (Peels, 2023) and yet we would not ordinarily ascribe ignorance to them^17^. In particular, the argument focuses on the following three strands of ignorance attributions exemplified by (A-C):

(A) ‘Giovanna is ignorant of the fact that there are ten thousand blades of grass in her lawn’.
  (attribution of ignorance about pointless truths)
(B) ‘Neoclassical artists were ignorant of the fact that Greek ancient statues and temples were painted in bright colours’.
  (attribution of ignorance about practically unknowable truths).
(C) ‘I am ignorant of the position of the particle X^18^’. [when I know its momentum]
  (attribution of ignorance about structurally unknowable facts).

The diagnosis offered is the following. (A-C) sound odd, or, if you want, ordinary speakers are not inclined to attribute ignorance in these cases, because those attributions would entail an unfair negative assessment of the agents in question. Attributing ignorance that *p* to someone entails that the agent has manifested a shortcoming, a failing in attaining the epistemic good they are lacking. Since no failure is at issue in these cases, (A-C) sound uncharitable at best, and are false at worse. In fact, in all three cases, the lack of a positive epistemic state with respect to *p* is not due to the agent’s intellectual failure. In the first case, we have the intuition that there seem to be no reason why Giovanna ought to have known the number of blades of grass in her lawn: this is a fact she is not expected to have inquired about. In the last two cases the agents couldn’t have known due to either practical or structural limitations (*a fortiori*, it is not the case that they ought to know those facts). We are not likely to say that neoclassical artists were ignorant of the fact that ancient Greek statues were painted in bright colours, because the historical sources were not clear on the matter, and the ruins they had access to were undyed—even if it could easily have been otherwise. Relatedly, we are not inclined to ascribe ignorance concerning propositions which are beyond the subject’s reach for structural reasons^19^, such as the subject’s epistemic limitations in (C).

According to the argument, the fact that statements (A-C) sound odd is best explained by the fact that the notion of ignorance we usually deploy has a normative component that has, until now, not been acknowledged. This means that we need a new account of ignorance that captures the intuition that ignorance is not only the absence of a valuable epistemic state, but something that carries negative normative force in its own right. Defenders of the so-called ‘Normative Account’ differ in how they articulate this normative dimension. Meylan (2020, 2024), for instance, employs axiological terminology, treating ignorance as a deficiency that possesses *prima facie*, non-instrumental disvalue. She has variously explicated this disvalue as instantiating the epistemic vice of insouciance (2020), as arising from a missed opportunity to know (2024), or—in more recent work with Reuter (2025)—as constituting a violation of legitimate expectations regarding what the agent ought to know. Pritchard consistently deploys a deontic language, instead (cf. 2016). Lack of knowledge that *p* counts as ignorance only if it manifests an intellectual failing on the agent’s part, and this hinges in turn on whether *p* was accessible for the agent (such that they were in a position to know it), on whether the agent has good reasons (epistemic as well as practical) to get to know that *p*, on whether the agent was already engaged in inquiry concerning the question whether-*p* (see e.g. Pritchard, 2022a, 2022b). For present purposes and given that both authors ultimately ground these normative considerations in the norms governing inquiry^20^, I will treat the Normative Account as a unified position characterized as follows. According to the Normative Account, given an agent S and a proposition *p*, S is ignorant that *p* if and only if,

(i) S doesn’t know/have a true belief that *p*, and.
(ii) this lack is due to the agent’s failure as an inquirer^21^
  (normative condition).

As one can see, the Normative Account is orthogonal or supplementary to the non-normative views on the nature of ignorance. Whichever positive epistemic value is lacking, this lack counts as ignorance only when it manifests an intellectual failure on the agent’s part. On the Normative Account one counts as ignorant of a fact only when one doesn’t know (or lacks a true belief about) a true proposition one ought to know. The normativity at issue here is the one of proper inquiry. At a very rough approximation, norms of inquiry regulate the pursuit of the answer to a question^22^. They prescribe which question is worth asking, how to seek the evidence which would be required to settle the matter, when it is appropriate to start and to conclude the inquiry, and so on and so forth. Endorsing the Normative Account of ignorance doesn’t commit one to taking a particular stand on what a proper inquiry should look like. Among the proponents of the Normative Account, for instance, Meylan understands norms of inquiry as distinctive epistemic norms, while Pritchard’s notion of inquiry is broader, including both epistemic and non-epistemic (e.g. prudential, moral) norms. Here it would suffice to say that according to the Normative Account, we are ignorant when the lack of knowledge or true belief of a given proposition results or manifests a breach of the above-mentioned norms of inquiry. To go back to cases (A-C), if one accepts the Normative Account, one would be able to explain why attributing ignorance in these cases would not be appropriate. In the first case, in fact, the question of how many blades of grass there are is not one worth pursuing; that’s why it is not the case that the agent ought to pursue it. In the second and third cases, the agents cannot even know that there is a conclusive answer to those questions. Since these agents cannot know the answers, it’s not the case that they ought to know them. In conclusion, we hesitate to attribute ignorance to them because they have not failed as inquirers, namely they have not conducted an improper inquiry.

Before turning to the positive part of the paper, let me briefly recap the key steps of the argument. According to the Argument from linguistic data, we are not inclined to attribute ignorance in cases where the agent lacks knowledge of pointless truths or of truths that are structurally or practically unknowable. The reason is that ascriptions of ignorance necessarily carry a negative assessment of the agent’s intellectual performance. This, in turn, is taken to support the view that ignorance has an inherent normative dimension: S is ignorant that *p* only when they lack knowledge or true belief that *p* as a result of a failure of proper inquiry. In what follows, I will develop an alternative explanation of the relevant linguistic data, one that undermines their evidential force in favour of the Normative Account of ignorance.

##  A pragmatic explanation of the normativity of ignorance attributions

In what follows, I am going to sketch a preliminary^23^, hopefully plausible picture of the communicative functions of ignorance attributions which freely borrows from the literature on the epistemology of ignorance^24^, feminist epistemologies of ignorance^25^, and agnotology^26^. Drawing on the vast literature on knowledge ascriptions^27^, I take a communicative^28^ function to be understood as the systematic role that a class of utterances—such as ignorance ascriptions—reliably performs in discourse, as revealed by recurring patterns of exemplary cases. While establishing the bearing of any of these functions on the semantics of ‘ignorant that *p*’ and more so on the definition of the concept of ignorance would require a principled argument, here I will only identify those functions via a heuristic approach. Hence, the picture is going to remain incomplete and not fully systematic: I don’t mean to exclude that ignorance ascriptions may fulfil other communicative functions, nor that some of these functions may be reduced or be parasitic on one another^29^. For the purposes of my argument, it will suffice to show that expressing criticism towards the agent as an inquirer is just one function among many and as such it doesn’t license any strong conclusion about the truth-conditions of attributions of ignorance^30^ and, *a fortiori*, about the nature of ignorance. I take these functions to pertain to the pragmatics of ignorance attributions and not to their semantics. Therefore, by demonstrating that ignorance attributions serve various communicative functions (excusing, criticizing, encouraging, etc.), I aim to show that not all of these functions can be reduced to implications of epistemic evaluation, at least not in the sense presupposed by the Argument from linguistic data in support of the Normative Account.^31^.

This discussion will support my argument that ‘being the result of a failure of inquiry’ is just a conversational implicature triggered by attributions of ignorance which are used to assess or criticize the agent as an inquirer. In § 3.2 I will proceed by arguing that in those kinds of conversational contexts, the utterance of ‘S is ignorant that *p*’ in conjunction with the Gricean maxim of Relevance (1975) triggers the implicature that *p* is something that the agent ought to know, thus that their ignorance is a failure of inquiry. Having this basic pragmatic machinery in place, I am going to put forward a pragmatic explanation of the linguistic data that are used to motivate the Normative Account in §3.3.

### Some communicative functions of ignorance attributions

Attributions of ignorance are speech acts which belong to the assertive family. They can serve many different purposes in our interpersonal communication. Some functions are more standardized than others, and they surface in philosophical literature on ignorance. My aim here is to show that not all the communicative functions of ignorance attributions track failure of inquiry. As anticipated in § 2, my focus will be on attributions of propositional or factual ignorance which are expressed by the predicate ‘is ignorant’ and a declarative complement. Statements which express propositional ignorance will thus take the following form: ‘S is ignorant *that p*’ and ‘S is ignorant *of the fact that p*’.

#### Criticizing the agent’s epistemic performance

1. ‘Camilla is ignorant that the literature she’s using is outdated [in the context of a research seminar].^32^
2. ‘My family doctor is ignorant that ulcers are not caused by stress’ [in the context of a complaint about the medical care received].

Ascriptions of ignorance can be a means of criticizing the agent’s epistemic performance. Asserting (2), for instance, typically implicates that^33^ the doctor ought to know that ulcers are not caused by stress, and this lack of knowledge is a manifestation of an intellectual failure on their part. Since this is a fact relevant to their profession, they have an obligation to inquire into it and moreover, as an expert, this is a fact that they have easily access to. The fact that they don’t know that ulcers are caused by stress is then a result of the agent’s violation of norms of evidence responsiveness and evidence-gathering. Similarly, in the context of an academic talk, an assertion such as (1) as a way to highlight the limitations of the presenter’s literature review, is a means of expressing criticism about their research, the beliefs they formed on the basis of the results, the conclusions they drew and, ultimately, the testimony they provided to the audience.

In these cases, attributions of ignorance involve a negative evaluation of the agent’s epistemic performance, because ascriptions of ignorance such as (1) and (2) typically triggers the implicature that the agent ‘has failed as an inquirer’. Given that in this kind of conversational context in which the agent’s epistemic performance is in focus, epistemic norms and norms of inquiry more generally strongly shape the discursive expectations of speakers and hearers in this setting. Consequently, ascriptions of ignorance such as (1) and (2) are most naturally understood as serving to criticize the agents’ epistemic conduct. In line with the Gricean maxim of Relevance (1975) ascriptions of ignorance of this kind are typically taken to convey, by way of a conversational implicature, that the agent has failed to meet some epistemic obligation. it would be natural to have negative affective reactions to such attributions of ignorance, and to provide excuses that would cancel the implicature.

While I will bolster my case and say more about the pragmatic mechanisms involved below, it is important to note here that this first function is precisely the one which is taken to be central in the Argument from linguistic data in support of the Normative Account of ignorance. In what follows, I will move on to other communicative functions of ignorance ascriptions which may undermine the linguistic argument for the Normative Account.

#### Excusing or justifying actions

(3) ‘The defendant was ignorant of the fact that the laptop was stolen’. [in the context of a courtroom]
(4) ‘I was ignorant that you were allergic to peanuts’ [in a conversation about an allergic reaction caused by peanuts].

In most conversational contexts, ascriptions of ignorance such as (3) and (4) are plausibly used to justify or at least excuse the agent for violating a given legal or moral norm: they have exculpatory value. In fact, whether someone is ignorant of a fact can have an impact on whether they can fully be held responsible for a given action and, ultimately, whether they should be blamed or praised for it^34^. Regarding (4), if I was ignorant of your peanut allergy, maybe I cannot be (entirely) blamed for giving you a donut which contains traces of peanuts, and for the catastrophic allergic reaction which follows. Importantly for our purposes, whether ignorance can fully excuse hangs on whether it is reasonable for the agent to be unaware of a given piece of information^35^. The exculpatory value of (1), for instance, would be very much reduced if we came to know that the defendant bought the computer on a very dubious website, or that they didn’t ask for any receipt after their purchase. What counts as reasonable may vary with the context, but of course blameless ignorance^36^ has the highest exculpatory value.

On the non-normative views, ignorance is simply the absence of knowledge, and its relationship to responsibility depends on whether the ignorance is blameless or culpable. Not knowing can excuse (for example, failing to detect gas in a room when no reasonable means of detection were available) but not in cases where one ought to have known (for example, if one forgets that one had turned on the gas). By contrast, on Pritchard’s Normative Account^37^ ignorance is inherently culpable: it is not mere non-knowledge but a failure to meet one’s responsibilities as an inquirer. This means that in cases where one’s lack of knowledge is excusing, the agent’s state doesn’t qualify as ignorance at all but is rather blameless non-knowledge. Hence, while people often appeal to their lack of knowledge in order to avoid blame, they do not, and cannot coherently, appeal to ignorance itself, since ignorance, on this conception, always carries fault. While Pritchard recognises the revisionist implications of his account, this can’t be the whole story. At the very least, he owes us an error theory that explains why ordinary speakers are systematically mistaken when they talk about ‘blameless ignorance’ and when they use ascriptions of ignorance in an exculpatory way.

#### Encouraging collective inquiry/assessing the gaps of inquiry

(5) ‘In early 2020 the scientific community was still ignorant that (of whether^38^) wearing masks is an effective preventive measure’
(6) ‘While our results show that drug X is effective in the short term, we are still ignorant that (of whether) it has a long-term efficacy’ [in the conclusion of a scientific paper].

Third person and self-attributions of ignorance may be used in the context of a scientific paper or a policy-making report. They serve to express caution, highlighting the gaps in the research and the limits of the available results. In the context of a policy-making debate in the early 2020, when there were still not conclusive (evidence-based) results about the efficacy of wearing masks in public^39^, an ascription of ignorance such as (5) could have been used to recommend caution or to justify hesitance in the face of uncertainty. By the same rationale, attributions of ignorance can also have the directive force of a recommendation, by highlighting the future lines of research^40^. In academic contexts self-ascriptions of ignorance such as (6) function as indirect directives: they identify a gap while simultaneously positioning that gap as worthy of attention. The mechanism is pragmatic: by explicitly marking something as unknown within the context of completed research, the speaker implies that the question is both tractable and significant. Moreover, this pragmatic mechanism is sustained by the norms of scientific practice^41^: identifying knowledge gaps is a standard move for “creating a research space” (Swales, 1990) and motivating new research questions (Sandberg & Alvesson, 2011) At the end of a scientific paper or talk, an attribution of ignorance such as (6) may prompt the readers or the audience to address a question which has emerged from the authors’ research and still remains unaddressed.^42^ In this way, ignorance ascriptions uttered by a speaker in the relevant epistemic standing can make legitimate research opportunities salient to the broader community.

#### Flagging systemic issues

(7) ‘In the Seventies most people were ignorant of the fact that smoking kills you’.
(8) ‘Many students at a college level are still ignorant that the mean length of the clitoris is 16 mm’.
(9) ‘To this day, many Italian citizens are ignorant that their government committed war crimes in Ethiopia’.

Ascriptions of ignorance such as (7) are frequent in the literature on agnotology (Proctor, 1995), a subfield in the sociology of knowledge, which investigates how ignorance and doubt can be *strategically* cultivated and maintained among laypeople to further the economic agenda of private companies. A famous example is provided by the misleading data manufactured and spread by tobacco companies in the Fifties about the health dangers of smoking.

In the same vein, philosophy of race (Frye, 1983; Mills, 1997, 2015) and feminist epistemologies of ignorance (Shannon and Sullivan, 2007, Tuana, 2004) focus on how certain agnogenic practices manifest and perpetuate oppression (8,9). Critical epistemology emphasizes the structural dimension of ignorance: ignorance is not just seen as the result of a faulty inquiry of the individual subject but is the byproduct of social practices which uphold racial (and other forms of) domination (Bain, 2023; Martìn, 2021). Hence, the normative component of ignorance should not be exhausted in reference to the individual subject’s agency^43^, but rather it should be assessed in reference to social institutions and practices that shape and constraint the individual’s epistemic environment (Alcoff, 2024).

#### Flagging unreliable informants

(10)  ‘Walter is ignorant that the cost-of-living crisis is happening!’ [in the context of a chat about the affordability of living in London].

Just like attributions of knowledge are used to identify reliable informants^44^, attributions of ignorance can be used to flag unreliable informants. If I tell you that our common friend is ignorant that the cost-of-living-crisis is happening, I can warn you against believing what they are going to say next about how affordable is to live in London (10).

As I have explained above, this cursory presentation of the communicative functions of ignorance is very tentative, and I don’t mean to exclude that some functions may be subsumed or reduced to others. For instance, this function #5 may be reduced to or subsumed under function #1 (criticizing the agent’s epistemic performance)^45^. There are two reasons why I think it is worth highlighting it separately here. First, while attributions of ignorance such as (10) still may be used to criticize the agent’s epistemic performance, this is not the performance that is relevant to ignorance (according to the normative view). In fact, in this case itis the normativity of assertion and testimony which are in focus, and not the normativity of inquiry. Relatedly, I think the illocutionary force is different: (10) seems to count primarily as a warning rather than a criticism. When uttering something like (10) in the kind of conversational context outlined above, what is conveyed is, plausibly, that Walter is not a trustworthy speaker, and there are sound reasons to dismiss any further information he may share and not to ask questions.

#### Warning against or for a given course of action

(11)  ‘You are ignorant that banks may have changed their opening hours for the holiday season.’ [in the context of deciding when to go to the bank].

As we already saw with (10), attributions of ignorance can have directive force^46^. They can be used not only to assess an agent’s actions and epistemic performance, but also to encourage or discourage them from taking a given course of action. Asserting (11) may for instance be a way of prompting you to check the bank opening hours again.

To sum up, we have just seen that we use ignorance ascriptions to serve a number of communicative functions, and that not all of them track failure of inquiry. If you think that the picture I have just sketched is plausible, then you have a *prima facie* reason to think that a pragmatic explanation of the normative weight of ignorance attributions is viable. In fact, the Argument from linguistic data in support of the Normative Account seems to assume that signalling an epistemic failure on the part of the agent is the constitutive or at least the most prominent communicative function of ignorance attributions, namely the one function to which all the others can be reduced or subsumed under. However, we have seen above that there are many perfectly plausible functions of attributions of ignorance which don’t carry any negative evaluative judgement on the part of the agent (#2, #3, #6) or do so (at least partially) insofar as the agent is part of a malfunctioning epistemic environment (#4). In this way, proponents of the Normative Account who want to motivate their view by appealing to the Argument from linguistic data, will also immediately have to resort to an error theory as to why the speakers are systematically mistaken when they attribute ignorance in a range of cases. This is a serious setback for champions of the Argument from linguistic data. If anything, once we consider the full gamut of functions that attributions of ignorance can serve, there is a *prima facie* reason to think that the normative weight of ignorance attributions is just a pragmatic phenomenon pertaining to some uses of ignorance ascriptions. Before proceeding to explaining the intuitions about the cases of ignorance attributions that motivate the Normative Account on a pragmatic level, let me introduce the final element of my argument: conversational implicatures.

### What is said and what is implicated: implicatures and proper usage

Conversational implicatures are, roughly, propositions that are not part of the semantic content of what is said nor logically implied by it but are part of the “conveyed meaning”^47^ of an utterance in a given conversational context. By way of example, if I ask you whether you have beer in your fridge and you reply by saying ‘I have quit drinking’, it is conversationally implicated that you don’t have any beer in your fridge. Within a broadly Gricean framework, conversational implicatures can be generated by either abiding by the conversational maxims (observation implicatures) or violating them (exploitation implicatures)^48^.

Conversational maxims can be summarized by the overarching Cooperative Principle, which prescribes to «make your conversational contribution such as is required, at the stage at which it occurs, by the accepted purpose or direction of the talk-exchange in which you are engaged»^49^. They can be understood as both normative expectations shared by the speaker and the hearers and as inferential principles which guide and constraint the hearer’s understanding of the speaker’s contribution to the conversation^50^. What is implicated is not just a matter of the speaker’s private communicative intentions (Sanders, 2013; Saul, 2002) but also (and even primarily) of what a generic speaker from the speaker’s and the hearer’s community would have intended by producing that utterance in that kind of context (“generic speaker meaning”^51^). In other words, assuming that the speaker is taking seriously the hearer’s informational needs, and that they are being cooperative either at the level of what is said or at the higher level of what is communicated^52^, the hearer should be able to work out quite easily what is being implicated by that utterance^53^. When I reply to your question that I have quit drinking, you almost immediately and without any conscious effort pick up on what is implicated: there is no beer in the fridge.

In addition to being calculable, genuine conversational implicatures must pass the so called “cancellability test”^54^, meaning that they can be cancelled by adding more information to the conversation, without thereby retracting the first assertion or incurring any contradiction. Sticking to the example above, I can add: ‘But there is some of my sister’s beer in the fridge’, thereby blocking you from inferring that there is no beer in the fridge. Based on how much the details of the occasion of use matter, a given conversational implicature may be more or less ‘regularized’ (Simons, 2012). At one end of the spectrum, particularized implicatures rely the most on the specific features of the conversational context and on the encyclopaedic knowledge shared by the conversational partners. Saying something like ‘you know my flatmate’ may only make available the proposition that ‘there is no beer in the fridge’ to a hearer who already knows that my flatmate typically steals all the beer I stack in our fridge.

‘Generalized’ or ‘general’ implicatures^55^ lie at the other end of the above spectrum. As a rule of thumb, the more a given implicature is regularized^56^, the more it is going to be difficult to cancel it. Generalized implicatures are generated in most contexts (albeit not in all) and, absent special circumstances, they are communicated with ‘what is said’ to the point that sometimes they can only be meta-linguistically cancelled. Going back to a slightly modified version of the beer vignette, if you asked me, ‘Can you pass me the beer?’, it would be very difficult, albeit not impossible, to cancel the implicated content that I am requesting for you to hand me a pint. It could be done by suitably specifying that the conversational context is not a typical one (e.g. the sentence is uttered during a physical therapy session) or by explicitly referring to one’s own utterance (e.g. ‘I said “Can you pass me the beer?” but *what I meant* is “Are you able to be nice to me tonight?”). The conditions of proper assertability of propositions are not only responsive to semantic considerations but also to pragmatic regularities such as the ones highlighted above. In fact, intuitions about the proper usage of a linguistic expression are both responsive to its conventional meaning and to the generic speaker meaning that those expressions generate in typical conversational contexts^57^. Consequently, when confronted with an odd assertion, it is often not immediately clear whether the oddity should be traced back to the truth conditions of the proposition asserted or to the truth conditions of the proposition pragmatically imparted in that (kind of) conversational context.

### Back to linguistic data: a pragmatic explanation of Pritchard’s cases

When discussing the cases that are used to motivate the Normative Account it is assumed that ordinary speakers refrain from attributing ignorance in those cases because they would be asserting something false. However, as stressed above, both semantic and pragmatic factors bear on conditions of proper assertability. Therefore, it is worth exploring the possibility that ordinary speakers are disinclined to make ignorance attributions in cases of lack of knowledge about pointless truths and structurally or practically unknowable truths because, albeit true, they would be infelicitous^58^. Let’s go back to the cases by focusing on one example for each.

(12)  ‘You are ignorant that the number of laboratory vials is even’.
  (ignorance of pointless truths)
(13)  ‘You were ignorant of the fact that (of whether) anyone has ever had these results before’.
  (ignorance of practically unknowable truths)
(14)  ‘You are ignorant that the particle’s position is X^59^’ [while knowing its momentum]
  (ignorance of structurally unknowable truths).

In a conversational context in which the epistemic performance of the subject is the central focus of the discussion, the attributions of ignorance above can be expected to be used to criticize the subject’s epistemic performance and to signal their failure as an inquirer. In fact, in such a context, given their salience, norms of inquiry and related expectations play a huge role in determining the conversational expectations of the interlocutors. If the speaker is being cooperative at the level of what is said, and they are abiding by the Gricean maxim of Relevance, which prescribes to make one’s contribution relevant to the goals and the scope of the conversation, they are making available the generic speaker meaning that the attributee lacks a positive epistemic good they *ought* to have—otherwise why mention it? In other words, uttering something like (12–14) in such conversational contexts may typically trigger the conversational implicature that the agent has failed as an inquirer.

If I am right here and the proposition *that you have failed as an inquirer* is neither expressed nor entailed by (12–14), we should expect at least two things. First, this implicature should not arise when the above attributions of ignorance are used to perform communicative functions other than criticizing the agents’ epistemic performance. Second, and relatedly, one should be able to cancel the implicature by adding more information to the conversational context. I will argue that both expectations are met. Think, for instance, of a conversational context in which attributions of ignorance about structurally or practically unknowable facts may be used to signal the limits of a given research project in a scientific paper or to excuse the agent’s action. Think, for instance, of a conversational context in which the agent published results that had already been published, slightly before, in a different journal, unbeknownst to them. Suppose that the agent couldn’t have gotten access to these facts and has been accused of plagiarism as a result. In such conversational context, saying something such ‘You were ignorant of the fact that (of whether) anyone had had these results before’ may make available the generic speaker meaning that the agent’s violation of deontological norms is excusable. What matters for the purposes of this paper is that it seems plausible that when attributions of ignorance are not used to fulfil the function of criticizing the agent’s epistemic performance, the proposition that *the agent’s state of ignorance is due to a failure of inquiry* is not imparted.

Finally, even in conversational contexts in which attributions of ignorance are used to criticize the agent’s epistemic performance, the implicature that they signal a failure of inquiry may be comfortably cancelled, either by adding more information to the common ground or by explicitly referring to it^60^ (saying something like: ‘You are ignorant of (the fact) that *p*, but I do not mean inquiry is flawed’). A conversation like the following, which involves the utterance of an attribution of ignorance about a structurally unknowable truth, for instance, doesn’t sound awkward.

(15) A: ‘Is your student ignorant that (of whether) Alexander the Great died of indigestion?’
  [*they ought to know it*,* their ignorance manifests a failure of inquiry*]
  B: Yes, but it this doesn’t mean that their research is flawed. There is not conclusive evidence in the ancient texts to ascertain that.

But suppose one remains unconvinced that ignorance attributions constitute epistemic criticism simply in virtue of being made in contexts where epistemic norms are salient. Or suppose that my piecemeal, incomplete outline of the communicative functions of ignorance attributions is not compelling. Does a pragmatic diagnosis of the cases that motivate the Normative Account still stand a chance? I think so. Let’s start with attributions of ignorance about pointless truths. Attributing ignorance about a fact that, *by stipulation*, has no significance in the current conversational context amounts to a non-observance of the same maxim of Relevance mentioned above. In the absence of more information, it is not clear to the hearer whether the speaker is not abiding by the Maxim in order to mislead them (violation), or they are intentionally and overtly flouting it in order to impart an implicature or, finally, whether the speaker’s failure to abide by the maxim is just the result of a wanting linguistic performance (infringement)^61^.

What about attributing ignorance about practically or structurally unknowable facts? The only contextual information that we have is that it is practically or structurally impossible to have access to the facts about which ignorance is predicated. A generic hearer with this background knowledge will find both (13) and (14) redundant, unnecessary, and they will wonder, again, how to interpret the speaker’s failure to abide by the Gricean Maxims of Quantity, which prescribe to make one’s own contribution as informative as is required for the current purpose of the exchange. Again, by enriching the context of utterance, the kind of failure may be apparent, and cooperative communication may be restored, for instance by mutual understanding that the Maxim is being flouted on purpose to convey a different implicature. The utterance of (15) in a conversational context where we are talking about your historical knowledge may for instance implicate that the students knows every detail of Alexander the Great’s biography, apart from what is contingently impossible to know.

Before turning to potential objections, it is worth summarizing the key steps of the argument presented so far. The linguistic argument for the Normative Account proceeds from observed patterns in ignorance attributions: ordinary speakers systematically avoid attributing ignorance in cases involving pointless truths or structurally/practically unknowable truths. Proponents take this datum as evidence that ignorance involves more than mere lack of knowledge, with the normative condition explaining why these cases fall outside the scope of genuine ignorance. In this section, I have argued that the Argument from linguistic data in support of the Normative Account relies on the premise that the primary (maybe even constitutive) function of ignorance attributions is negatively assessing the epistemic performance of the agent. Here I have cast doubt on this premise, by showing that expressing epistemic criticism is just one communicative function of ignorance attributions among many and that ascriptions of ignorance in those cases, albeit true, are infelicitous. In fact, either the proposition *that the agent ought to know p* is just a conversational implicature of ignorance ascriptions used to assess the agent’s epistemic conduct, or the hearer does not have enough information to work out the speaker meaning of those utterances. In conclusion, I hope to have provided enough evidence to support the claim that the normative import of ignorance attributions can be satisfactorily explained by pragmatic mechanisms.

## Two potential worries considered

Before concluding, I will briefly lay out the implications of this argument and address two potential worries. First, am I committed to the claim that the conventional meaning of ‘S is ignorant that *p*’ is the same as the conventional meaning of ‘S doesn’t know that *p*’? Once the normative condition is out of the way, can we conclude that attributions of propositional ignorance and negative attributions of propositional knowledge have the same truth conditions? The short answer is ‘no’. While my pragmatic explanation favours the non-normative views, it doesn’t help to settle the dispute between the so-called Standard View (ignorance as lack of knowledge) and the New View (ignorance as lack of true belief). Hence, it still leaves open the possibility that ignorance is not the polar opposite of knowledge^62^.

Nonetheless, I recognize that this manoeuvre might appear unsatisfactory. Be as it may, if I were right, one may still wonder whether the same conversational implicature arises for negative attributions of knowledge and/or true belief. But consider for instance:

(16) ‘Giulio is ignorant that Paris is the capital of France, but of course there is nothing wrong with that.’
(16’) ‘Giulio doesn’t know/doesn’t have a true belief that Paris is the capital of France, but of course there is nothing wrong with that.’

Arguably, (16) sounds worse than (16’)^63^. The Normative Account can explain why. Can the pragmatic view? I will here offer an attempt at an explanation. Consider now the following utterances:

(17) ‘What you said yesterday is false, but I understand’.
(17’) ‘What you said yesterday is not true, but I understand’.

While both utterances carry (in most ordinary contexts) the implicature that ‘you shouldn’t have said what you said’, and they may be both used to express criticism, in (17’) the criticism is mitigated by the straightforward negation of the antonym adjective (Leech, 2014, p. 193). As it is emphasized in the scholarship on the pragmatics of politeness (see e.g. Kádár & Haugh, 2013 for an overview) when making a conversational contribution speakers are not only interested in getting their message across, but also in enhancing and protecting their social status (‘facework’ or relational work). In a nutshell, I think that the awkwardness of the implicature cancellation in (16) and (17) must be traced back to pragmatic mechanisms of politeness. Very roughly, a hearer who is confronted with those utterances would wonder why a speaker who doesn’t want to implicate that the agent they are criticizing is blameworthy (in fact, in both the examples this implicature is immediately cancelled) chooses to express their criticism in such an undiluted, strong way.

At this point, an unconvinced reader may ask: why not go contextualist about ignorance attributions? Why not take seriously the intuitions elicited by the Pritchard’s cases and argue that norms of inquiry set the contextual threshold for true attributions of ignorance? Here I will just note that such a strategy^64^ would still need to be complemented with an error theory which explained why we are systematically mistaken when attributing ignorance in cases such as (3–6). Moreover, a contextualist strategy applied to ignorance attributions would likely hang on ‘ignorant’ being a relative gradable adjective. As far as I know there is no consensus in the literature as to whether ‘ignorant’ is a partial or an absolute gradable adjective. If the latter turned out to be right, the adjective ‘ignorant’ would not have a place for a comparison class and it would not (at least automatically) afford a contextualist semantics. In fact, if ‘ignorant’ turned out to be an absolute gradable adjective such as ‘pure’, ‘straight’ or ‘flat’ (Siscoe, 2024)^65^, it would be appliable to every agent who is ignorant to some degree, and it would only leave out agents who don’t possess this property at all: agents who know/have a true belief that *p*.

## Conclusion

Attributions of ignorance often carry normative weight. I have argued that this phenomenon is better explained on a pragmatic level by looking at implicatures of ignorance ascriptions in stereotypical contexts and at the communicative functions that they play in our day-to-day social interactions. A theory locating the normativity of ignorance attributions in pragmatic mechanisms is compatible with simple theories of the nature of ignorance. Moreover, it avoids the challenge of providing an error theory for non-normative ignorance attributions, such as attributions of blameless ignorance, ascriptions with exculpatory value, and self-attributions of ignorance.

As discussed in the previous section, my argument does not directly settle which account of the nature of ignorance, or the semantics of ignorance attributions is correct. However, accepting that the normative significance of ignorance ascriptions belongs to pragmatics, and that true ignorance attributions need not track failures of inquiry, represents an indirect step toward addressing these questions. Specifically, I have shown that there are no compelling grounds for accepting the Argument from linguistic data in favour of the Normative Account. Other arguments for incorporating a normative condition into our account of ignorance may yet prove successful, and the debate remains open.

More broadly, this paper contributes to a larger project within the epistemology of ignorance. While ignorance itself has only recently become a central topic in analytic epistemology, existing work in agnotology, moral philosophy, and feminist epistemologies of ignorance provides a valuable foundation. Engaging with these perspectives requires considering not just how ignorance and its ascriptions are used to evaluate an agent’s epistemic performance (Piedrahita, 2025a) but also what other communicative functions they serve. A focus on the normative dimension of ignorance attributions as a means of criticizing the epistemic performance of agents alone may suggest drawing conclusions that are disconnected from broader epistemological discussions in these fields. If correct, my pragmatic account of the linguistic data offered in support of the Normative Account may help bridge this gap, by clarifying how ignorance ascriptions may operate within different theoretical frameworks.

## Footnotes

1 See for instance Willard-Kyle (2024) for an endorsement of this account within the inquiry literature.

2 For a similar argument (from warranted assertability) against contextualism with respect to knowledge attributions see Brown (2006), Brown and Gerken (2012), Rysiew (2001). For an assessment of the efficacy of such a move in that debate see Blome-Tillmann (2022), Dinges (2016) and Gerken (2017).

3 Cf. Williamson (2000), Zimmerman (2008).

4 Cf. Guerrero (2007, pp. 62–63).

5 For a discussion of the Normative Account and why it doesn’t sit well with blameless ignorance see Piedrahita (2025b). While my discussion here stems from linguistic considerations, that paper explores the metaphysical implications of the Normative Account and thoroughly explores cases of faultless suspension and blameless ignorance.

6 What exactly is meant by ‘normative’ will be clarified in § 2 below.

7 I use here the taxonomy provided by Nottelmann (2016); for a discussion of the notion of erotetic ignorance see Rescher (2009).

8 According to Le Morvan (2022, p. 173 and ff.) ignorance of a proposition consists in the incapacity to grasp the meaning of a proposition and factual ignorance (ignorance *that* p) consists in not knowing the truth value of a proposition that one grasps (see e.g. Le Morvan & Peels, 2016). This specification is important because Le Morvan concedes that one can be *factually* ignorant of false propositions they grasp, namely that *factual* ignorance is not factive. For instance, I might not know whether “there are fairies in my garden” is true or false, and thus be factually ignorant about it, even though the proposition is false. However, the other proponents of the Standard Account (namely the view that Ignorance equals lack of knowledge) do not agree with Le Morvan on this. This will not be relevant for the purposes of this essay. The disagreement between the Standard and the New Account of ignorance has centred on cases where agents who have a true belief which doesn’t clearly amount to knowledge can be deemed ignorant (e.g. gettierized subjects). For a critique of the Standard View which also addresses this difference see Piedrahita (2021). For a different account of ignorance see Carter and Piedrahita (2025), De Nicola (2017), El Kassar (2018), El Kassar (2025), Piedrahita (2025a), Silva and Siscoe (2024).

9 cf. Nottelmann, 2016 for a taxonomy of ignorance according to its object, degree and order.

10 Being ignorant (of the fact) that p can, although it needn’t necessarily, issue in, or manifests itself in various attitudes (or lack of thereof) towards the proposition that *p*, such suspending judgement, disbelieving, failing to consider and so forth (see Nottelmann, 2016 and for a useful review and taxonomy of the extant literature see Peels, 2023, pp 74–97). In other words, propositional ignorance can also come in different forms, and different syntactic constructions may be more appropriate in one case or in another. However, since this goes beyond the scope of my current analysis, I will not be making use of these distinctions here.

11 The sake of simplicity I will deal with the limit case of ignorance about a single proposition unless otherwise specified.

12 See e.g. Brogaard (2016, p. 58) and Unger (1975, p. 175). One might also consider ‘S ignores (the fact) that p’ as an alternative, admittedly much more used, construction. In this case one should also appreciate the difference between the sense of ‘ignoring that p’ that implies failing to know that p and the sense of ‘ignoring that p’ that does not imply failing to know or at least being aware of p, as in ‘In your reasoning you ignore the fact that the elections are in two years’. In fact, ‘to ignore’ is standardly understood as an act of intentionally disregarding or refusing to consider something one is aware of (see e.g. Lewis 1996 ). This means that the conditions under which someone ignores that *p* and those under which someone can be truthfully deemed ignorant that *p* differ (Meylan, 2024, § 3.4). The conceptual relations between ‘ignorance’ and ‘ignoring’ are widely explored in the literature on ‘active ignorance’, see e.g. Dotson (2011, p. 243), El Kassar (2018, 2025), Medina (2013, p. 39).

13 The authors report example from the *News on the Web English Corpus* (pp. 4–5). A rapid search on the *Corpus of Contemporary American English* reveals 77 occurrences of ‘ignorant that’ and 42 occurrences of ‘ignorant of the fact that’.

14 I am also sympathetic to the suggestion made by Silva and Siscoe (2024, p. 234) that ‘ignorant that-’ constructions could be a contraction of ‘ignorant of the fact that-’ constructions.

15 Peels (2023, pp. 26–27); cf. Kyle (2015, 2021).

16 See Nottelman (2016), and Peels (2023, pp. 35–36). However, this is not entirely uncontroversial, see e.g. Piedrahita (2025a, fn. 5), Rescher (2009, pp. 28–29) and Wilholt (2020, p. 199).

17 While the examples are mine, except from the one concerning the position of the particle, these three strands of cases are discussed by Pritchard (2021a, 2021b) and defended by Meylan (2020, 2024). Pritchard also discusses a fourth strand of cases regarding attributions of ignorance of hinge propositions, namely propositions that are not ‘in the market for knowledge’ to start with. Since this fourth strand of cases only appeals to those who already subscribe to hinge epistemology, I will not address it in the remainder of the paper. For a discussion of these cases and the Normative Account of ignorance see Piedrahita (2025b), Wang and Wang (2023).

18 This example is from Pritchard (2021b, p.5). This example may be confusing, as, according to some views in the metaphysics of Physics, there would be no fact of the matter. Since it is not established in contemporary Physics whether the particle has a position, but it is unknowable or whether it is ontologically indeterminate, here I take the point is that there is a fact of the matter, but it goes beyond the subject’s reach. Thanks to an anonymous reviewer for helping me to clarify this point.

19 Cf. Rescher (2009) for examples of things we are necessarily ignorant about.

20 See e.g. Meylan (2024, p. 14; 2020, pp. 443–446), Pritchard (2021a, 2021b, 2022b). Many thanks to an anonymous reviewer for helping me being clear about this.

21 I use this wording here instead of ‘failure of inquiry’ to forebear the implication that the normative condition may be met in cases where the inquiry fails due to external factors which have nothing to do with the subject’s epistemic agency (such as their death). On the topic of the connection between ignorance and epistemic agency, see e.g. Piedrahita and Carter (2025). When, for simplicity, I will keep using the latter phrasing through the paper, I will intend it in the sense explained here.

22 For a helpful summary of the recent literature on the epistemology of inquiry see Haziza (2023) and Falbo (2025). To have an idea of how the norms of inquiry ‘interact’ with the more traditional norms of belief see Friedman (2020) and Kelp (2021).

23 While much philosophical and experimental work has been devoted to investigating attributions of knowledge, the philosophical work on attributions of ignorance—at least as something distinct from negative attributions of knowledge—is just now beginning see e.g. Meylan and Reuters (2025). While relating my contribution to their work would exceed the limits of this paper, their two-senses account of ignorance that encompasses both a normative and a non-normative dimension is entirely compatible with my analysis below. Much has already been written about negative attributions of knowledge, see for instance Vollet (2020), Blome-Tillmann (2016), Stoutenburg (2017). Drawing directly from this literature would of course amount to begging the question, as what is at stake here is precisely whether attributions of ignorance amount to something more attributions of lack of knowledge (or true belief).

24 E.g. Peels and Blaaw (2016), Peels (2023).

25 E.g. Sullivan and Tuana (2007), Mills (1997, 2015) Tuana (2004), Frye (1983). The notion of ignorance has also been front and centre in the theorizing of hermeneutical injustice, see e.g. Bain (2015), Fricker (2016), Pohlhaus (2012).

26 The seminal work is Proctor (1995). For a recent example of how the agnotology framework can be applied in philosophy of science see for instance Fernàndez Pinto (2020).

27 For a classic example of the argument from functional role which, very roughly, is purported at establishing a semantics of ‘know’ and, relatedly, the concept of *knowledge*, drawing from the social function of attributions of knowledge see Craig (1990). For other examples of the so called ‘functional arguments’ in the debate about contextualism with respect to knowledge attributions see e.g. Hannon (2013, 2015), Henderson (2009, 2011), McKenna (2013, 2014). For a critical discussion of the argument from functional role see Gerken (2017, pp. 203–208). For some preliminary considerations on how to apply this functional approach to attributions of ignorance see Riegler (2024, 2025, pp. 73–74).

28 While I recognize that the term ‘communicative’ might suggest an undesired restriction on the sort of function served by a class of utterances, as if transmission of information were predominant, I choose this word instead of ‘conversational’ or ‘discursive’ because it is commonly used in the parallel debate on knowledge attributions (for a summary see Gerken, 2017, Ch.8).

29 Here I don’t want to cast out these as unimportant issues. One reason is that there may be functions that are not genuine in that they are not regularized enough, and they don’t elucidate interesting uses of attributions of ignorance in the context of this paper, such as the alleged ‘praising function’ of statements such as ‘She was ignorant that there was a reward for her good action’, which seems to signal a virtue (see e.g. Townley (2011) on the positive instrumental value of ignorance in relationships). While the work to be undertaken to properly elucidate the distinctive communicative functions of ignorance attributions and their bearing on the concept and the epistemology of ignorance is still much, I think it goes beyond the modest point I am trying to establish in this paper. Many thanks to an anonymous reviewer for pointing this out.

30 Cf. Gerken (2017, pp. 202–210).

31 Many thanks to an anonymous reviewer for asking me to clarify this point.

32 It may seem redundant to include this function in the list, given that the Normative Account already accommodates it. However, while my focus in this section is on communicative functions that appear incompatible with the claim that ignorance attributions necessarily involve negative evaluations of the agent, function #1 will prove crucial in the following section, where I offer a pragmatic explanation of the linguistic intuitions that are used to support the Normative Account.

33 Note that the evidence is not available in the sense that she doesn’t have to do anything to get it, but rather in the sense that she *ought* to have this evidence, because she should stay on top of recent developments in her field see e.g. Goldberg (2017) and Simion (2023).

34 See Rudy-Hiller (2018) for a broad introduction to this topic.

35 For a discussion on how blameless ignorance poses a problem for the Normative Account—which prescind from the current focus on ascriptions of ignorance see Piedrahita (2025b), and Wang and Wang (2023).

36 A classical reference is Rosen (2003). Of course, here I am limiting my considerations to the exculpatory power of factual ignorance as opposed to moral ignorance, see e.g. Harman (2011), Haas and Vogt (2015). For a discussion of how the Normative Account of ignorance fares with respect to wilful ignorance in the context of Anglo-American law see Bnefsi (2024).

37 Pritchard (2021b, pp. 6–7). For a discussion of how the Normative Account fares with respect to responsibility in Anglo-American legal frameworks see Bnefsi (2024).

38 The construction ‘ignorant whether-’ seems more appropriate in (5) and especially (6), given that the assertion is made from a standpoint of ignorance. Note that this is true especially if we use the present tense like in (6). In a case like (5), instead, one can also suppose that even if the attributor and the person to which ignorance is attributed coincide, the person knows that they were ignorant that *p* in the past. Moreover, given that in the taxonomy I am using erotetic ignorance can be reduced to propositional ignorance, not much hangs on whether (5) or (6) express the former or the latter.

39 A good example of this discussion is Greenhalgh et al. (2020).

40 See e.g. the discussion on the linguistics of ignorance in Janich and Simmerling (2022).

41 See e.g. Stocking and Holstein (1993) for a seminal linguistic study on ignorance claims in scientific communication.

42 Cf. the debate ignorance norm of inquiry in the epistemology literature started by Friedman (2017). Very roughly, the intuitive idea behind this is that it is not permissible to inquire into a question if you already have the answer. For a good summary of the debate, I will refer you again to Haziza (2023, § 2) and Falbo (2025).

43 Though see the discussion on *active* ignorance and how to remedy it within a virtue epistemology framework see the recent monograph by El Kassar (2025). For a useful discussion on motivated ignorance and wilful ignorance see e.g. Freedman (2024). This point also speaks to the argument recently made by Piedrahita (2025b) when talking about cases where ignorance stems from others obstructing the agent’s access to truths rather than from the agent’s own epistemic failing.

44 Cf. Craig (1990).

45 Equally someone (e.g. Craig, 1990) might think that function #1 reduces to this one; it’s not my goal in this paper to settle the matter.

46 Following Gerken (2017, p. 171) here I use the locution ‘directive force’ instead of speaking of ‘directives’ as Bach and Harnish (1979) do. What I mean here is simply that attributing ignorance to an interlocutor or, in general, an agent can be a means to recommend or discourage a given course of action .

47 See Lycan (2018, p. 163).

48 This terminology is from Dinges (2015). See Bianchi (2013) for an accurate explanation of the different kinds of exploitation implicatures.

49 Grice (1975, p. 26).

50 Importantly, the cognitive and the normative aspects of the Gricean maxims have been recently recognized as complementary, see e.g. Hu (2024).

51 See Sbisà (2023) but cf. Bianchi (2013) and Davis (2007) for a dissenting reconstruction of Gricean implicatures.

52 As the different varieties of non-abiding by the Gricean maxims show, not abiding by a maxim should not be equated to being a non-cooperative speaker, see Hu (2024, p. 116).

53 Whether the speaker is or should be held accountable for implicated content is a contested issue, see e.g. Mazzarella et al. (2018) and Meibauer (2023).

54 Blome-Tillmann (2013), Zakkou (2018).

55 See e.g. Grice (1975, p. 39) cf. Levinson, 2000.

56 The proposition that the agent’s lack of knowledge is the result of an improper inquiry is pragmatically not imparted by all attributions of ignorance (it is not a general implicature) nor it depends so much on the particular feature of a given conversational context (including the interlocutors’ background knowledge). I think this implicature is highly regularized, because it is triggered in a specific kind of conversational contexts, namely those in which ignorance ascriptions fulfil the function of assessing the agent’s epistemic performance cf. Gerken (2017, pp. 180–187).

57 See e.g. Bach (1995, 2008). For Warranted Assertability Manouvers against contextualism with respect to knowledge attributions see Brown (2006) and Rysiew (2001). For an assessment of the efficacy of such a move in that debate see Blome-Tillmann (2022), Dinges (2016), Gerken (2017) and Preyer (2010).

58 A similar point is made by Piedrahita (2025b, p. 2916). See also Peels (2023, pp. 65–67) for a competing explanation of the linguistic intuitions that are used to motivate the Normative Account.

59 Given that (14) is necessarily uttered from a standpoint of ignorance, it would be best be characterized as an attribution of ignorance with respect to the answer to a question (erotetic ignorance). See § 3.1.

60 Blome-Tillman (2013, p. 4). In this paper, the author articulates what he calls Principle of Cancellability (“If an utterance of P conversationally implicates q in C without semantically entailing it, then there is a context C# in which utterances of P do not commit the speaker to q”)and Principle of Explicit cancellability (“If an utterance of P conversationally implicates q in C without semantically entailing it, then utterances of P, but not Q” or P, but I don’t mean to imply that Q” are admissible in C and they cancel the speaker’s commitment to q”). One may argue that the fact that the implicature in question doesn’t arise when (12–14) are uttered in a different context is already sufficient evidence of their cancellability. However, for the purpose of clarity, here I want to explicitly distinguish a conversational context in which the utterances (12–14) dosn’t trigger the implicature in question, because the attribution of ignorance serves another communicative purpose, from a context in which the implicature arises and is cancelled. See also Blome-Tillman (2008).

61 For a thorough discussion on this see Hu (2024, p. 112).

62 See Kyle (2015, 2021) and Willard-Kyle (2024, pp. 1480); See also Piedrahita and Carter (2025) for a different take on why the notions of ignorance and knowledge are not polar opposites, which centres on epistemic autonomy.

63 Note also non-detachability cf. Blome-Tillmann (2013).

64 I don’t want to rule out that a contextualist strategy may turn out to be successful, but I think that the argument I made here makes it unlikely that a contextualist strategy along these lines may succeed. However, it is not immediately clear how it would improve on invariantism on one hand and, on the other hand, how it would be compatible with the Normative Account as outlined above.

65 A classical reference on relative gradable adjectives is Kennedy (2007).
